# Mindsets of Negative Emotions Mediate the Impact of Self-Compassion on Youth Mental Health: Evidence from a Three-Annual Wave Study

**DOI:** 10.1192/j.eurpsy.2025.490

**Published:** 2025-08-26

**Authors:** D. Qi, S. Zhu

**Affiliations:** 1 Department of Applied Social Sciences; 2 Mental Health Research Centre, The Hong Kong Polytechnic University, Hong Kong, Hong Kong

## Abstract

**Introduction:**

Accumulating evidence supports the mental health benefits of self-compassion. However, longitudinal research unravelling how self-compassion affects mental health outcomes is scarce.

**Objectives:**

This study aimed to clarify whether mindsets of negative emotions serve as a mechanism of how self-compassion exerts impacts on youth mental health.

**Methods:**

The longitudinal school-based survey was conducted annually across three years among 719 secondary school students (mean age: 14.65, SD: 0.72). Mediation models were examined with self-compassion at T1 as the independent variable, negative emotion mindsets at T2 as the mediator variable, and depression, anxiety, and life satisfaction at T3 as the dependent variables. Alternative mediation models with negative emotion mindsets at T1 as the independent variable and self-compassion at T2 as the mediator variable were also tested to corroborate the hypothesized direction of the mediation effects. The mediation models all controlled for age, sex, socioeconomic status (SES), and respective measurements of mediator and dependent variables at baseline.

**Results:**

Negative emotion mindsets at T2 significantly and fully mediated the effects of self-compassion at T1 on mental health (indexed by depression, anxiety, and life satisfaction levels) at T3 (see Fig. 1, Fig. 2, and Fig. 3, respectively). The alternative mediation models were all insignificant, corroborating that negative emotion mindsets serve as the pathway through which self-compassion impacts mental health, rather than the reverse.

**Image 1:**

**Figure:**
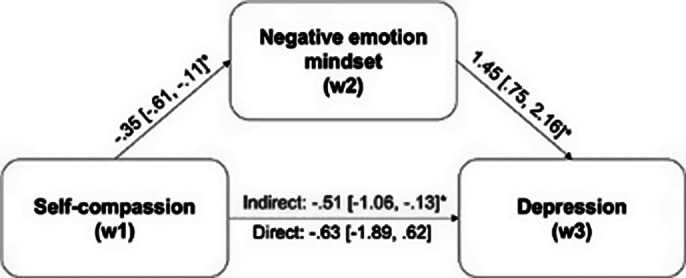

**Image 2:**

**Figure:**
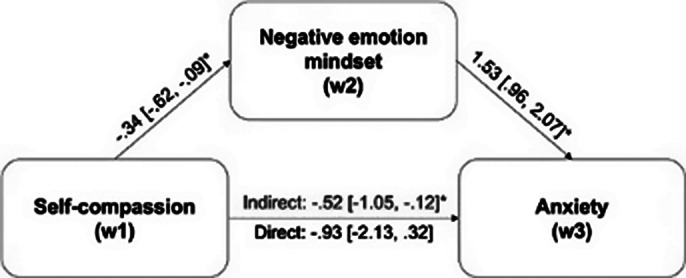

**Image 3:**

**Figure:**
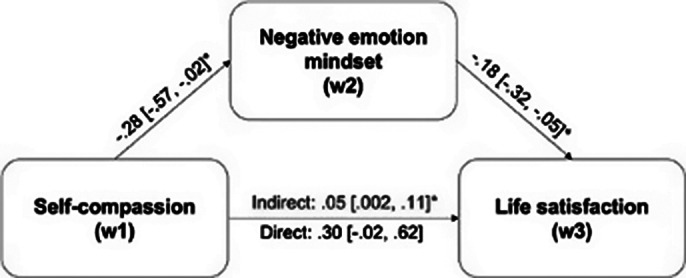

**Conclusions:**

This study highlights the belief-in-change of negative emotions as a key mechanism underlying the impact of self-compassion, and has important implications for research and interventions aimed at promoting youth mental health.

**Disclosure of Interest:**

None Declared

